# 2-(4-Fluoro­phen­yl)-1,4,5-triphenyl-1*H*-imidazole

**DOI:** 10.1107/S1600536810035464

**Published:** 2010-09-08

**Authors:** P. Gayathri, A. Thiruvalluvar, N. Srinivasan, J. Jayabharathi, R. J. Butcher

**Affiliations:** a PG Research Department of Physics, Rajah Serfoji Government College (Autonomous), Thanjavur 613 005, Tamilnadu, India; b Department of Chemistry, Annamalai University, Annamalai Nagar 608 002, Tamilnadu, India; c Department of Chemistry, Howard University, 525 College Street NW, Washington, DC 20059, USA

## Abstract

In the title mol­ecule, C_27_H_19_FN_2_, the imidazole ring is essentially planar [maximum deviation = 0.004 (1) Å] and makes dihedral angles of 62.80 (6), 36.98 (6), 33.16 (6) and 46.24 (6)°, respectively, with the substituent rings in the 1-, 2-, 4- and 5-positions. No classical hydrogen bonds are observed in the crystal structure.

## Related literature

For the synthesis and pharmacological evaluation of substituted 1*H*-imidazoles, see: (Nagalakshmi, 2008 ▶). For contact allergy to imidazoles used as anti­mycotic agents, see: Dooms-Goossens *et al.* (1995 ▶). For related structures and applications of imidazole derivatives, see: Gayathri *et al.* (2010*a* ▶,*b* ▶,*c* ▶).
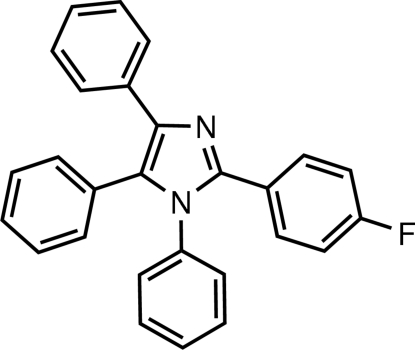


## Experimental

### 

#### Crystal data

C_27_H_19_FN_2_
*M* *_r_* = 390.44
Triclinic, 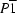

*a* = 10.1794 (5) Å
*b* = 10.5239 (6) Å
*c* = 10.6175 (6) Å
α = 80.750 (5)°
β = 85.776 (4)°
γ = 67.348 (5)°
*V* = 1035.95 (11) Å^3^
*Z* = 2
Mo *K*α radiation
μ = 0.08 mm^−1^
*T* = 295 K
0.51 × 0.44 × 0.15 mm

#### Data collection

Oxford Diffraction Xcalibur Ruby Gemini diffractometer
Absorption correction: multi-scan (*CrysAlis PRO*; Oxford Diffraction, 2010 ▶) *T*_min_ = 0.973, *T*_max_ = 1.000
15513 measured reflections
8350 independent reflections
3489 reflections with *I* > 2σ(*I*)
*R*_int_ = 0.030

#### Refinement

*R*[*F* ^2^ > 2σ(*F* ^2^)] = 0.049
*wR*(*F* ^2^) = 0.128
*S* = 0.86
8350 reflections
271 parameters
H-atom parameters constrained
Δρ_max_ = 0.22 e Å^−3^
Δρ_min_ = −0.19 e Å^−3^

### 

Data collection: *CrysAlis PRO* (Oxford Diffraction, 2010 ▶); cell refinement: *CrysAlis PRO*; data reduction: *CrysAlis PRO*; program(s) used to solve structure: *SHELXS86* (Sheldrick, 2008 ▶); program(s) used to refine structure: *SHELXL97* (Sheldrick, 2008 ▶); molecular graphics: *ORTEP-3* (Farrugia, 1997 ▶); software used to prepare material for publication: *PLATON* (Spek, 2009 ▶).

## Supplementary Material

Crystal structure: contains datablocks global, I. DOI: 10.1107/S1600536810035464/tk2707sup1.cif

Structure factors: contains datablocks I. DOI: 10.1107/S1600536810035464/tk2707Isup2.hkl

Additional supplementary materials:  crystallographic information; 3D view; checkCIF report

## Figures and Tables

**Fig. 1.**
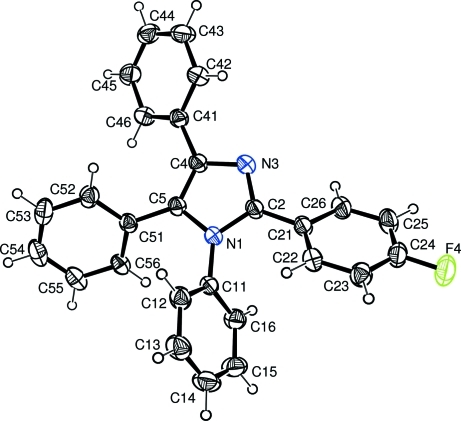
The molecular structure of the title compound, showing the atom-numbering scheme and displacement ellipsoids drawn at the 30% probability level. H atoms are shown as small spheres of arbitrary radius.

## supplementary crystallographic information

### Comment

Nagalakshmi (2008) has reported synthesis and pharmacological evaluation of 2-(4-Halo substituted phenyl)-4,5-diphenyl-1*H*-imidazoles, and Dooms-Goossens *et al.* (1995) have reported contact allergy to imidazoles used as antimycotic agents. As part of our research (Gayathri *et al.*, (2010*a*,*b*,*c*)), we have synthesized the title compound (I) and report its crystal structure here.

In (I), Fig. 1, the imidazole ring is essentially planar [maximum deviation = 0.004 (1) Å for N1]. The imidazole ring makes dihedral angles of 62.80 (6), 36.98 (6), 33.16 (6) and 46.24 (6) ° with the phenyl (C11—C16) attached to N1, fluorophenyl (C21—C26) attached to C2, and two phenyl rings (C41—C46) & (C51—C56) attached to C4 and C5, respectively. The phenyl ring at N1 makes dihedral angles of 54.26 (6), 85.21 (7) and 65.02 (6) ° with the fluorophenyl at C2, and phenyl rings attached to C4 and C5, respectively. The fluorophenyl ring makes dihedral angles of 63.01 (6) and 78.99 (6) ° with the phenyl rings at C4 and C5, respectively. Finally, the dihedral angle between the phenyl rings at C4 and C5 is 51.10 (6) °. In the crystal structure no classical hydrogen bonds are observed.

### Experimental

To benzil (3.15 g, 15 mmol) in ethanol (10 ml), aniline (1.5 g, 15 mmol), ammonium acetate (7 g, 15 mmol) and *p*-fluorobenzaldehyde (1.7 g, 15 mmol) were added over about 1 h while maintaining the temperature at 333 K. The reaction mixture was refluxed for 7 days and extracted with dichloromethane. The solid that separated was purified by column chromatography using hexane: ethyl acetate as the eluent. Yield: 3.51 g (60%).

### Refinement

H atoms were positioned geometrically and allowed to ride on their parent atoms with C—H = 0.93 Å, and with *U*_iso_(H) = 1.2*U*_eq_(parent atom).

### Crystal data

**Table:**

C_27_H_19_FN_2_	*Z* = 2
*M**_r_* = 390.44	*F*(000) = 408
Triclinic, *P*1	*D*_x_ = 1.252 Mg m^−^^3^
Hall symbol: -P 1	Melting point: 509 K
*a* = 10.1794 (5) Å	Mo *K*α radiation, λ = 0.71073 Å
*b* = 10.5239 (6) Å	Cell parameters from 3942 reflections
*c* = 10.6175 (6) Å	θ = 5.1–34.9°
α = 80.750 (5)°	µ = 0.08 mm^−^^1^
β = 85.776 (4)°	*T* = 295 K
γ = 67.348 (5)°	Plate, colourless
*V* = 1035.95 (11) Å^3^	0.51 × 0.44 × 0.15 mm

### Data collection

**Table:**

Oxford Diffraction Xcalibur Ruby Gemini diffractometer	8350 independent reflections
Radiation source: Enhance (Mo) X-ray Source	3489 reflections with *I* > 2σ(*I*)
graphite	*R*_int_ = 0.030
Detector resolution: 10.5081 pixels mm^-1^	θ_max_ = 35.0°, θ_min_ = 5.1°
ω scans	*h* = −15→12
Absorption correction: multi-scan (*CrysAlis PRO*; Oxford Diffraction, 2010)	*k* = −16→14
*T*_min_ = 0.973, *T*_max_ = 1.000	*l* = −17→16
15513 measured reflections	

### Refinement

**Table:**

Refinement on *F*^2^	Primary atom site location: structure-invariant direct methods
Least-squares matrix: full	Secondary atom site location: difference Fourier map
*R*[*F*^2^ > 2σ(*F*^2^)] = 0.049	Hydrogen site location: inferred from neighbouring sites
*wR*(*F*^2^) = 0.128	H-atom parameters constrained
*S* = 0.86	*w* = 1/[σ^2^(*F*_o_^2^) + (0.0586*P*)^2^] where *P* = (*F*_o_^2^ + 2*F*_c_^2^)/3
8350 reflections	(Δ/σ)_max_ = 0.001
271 parameters	Δρ_max_ = 0.22 e Å^−^^3^
0 restraints	Δρ_min_ = −0.19 e Å^−^^3^

### Special details

**Table:**

Geometry. Bond distances, angles *etc*. have been calculated using the rounded fractional coordinates. All su's are estimated from the variances of the (full) variance-covariance matrix. The cell e.s.d.'s are taken into account in the estimation of distances, angles and torsion angles
Refinement. Refinement of *F*^2^ against ALL reflections. The weighted *R*-factor *wR* and goodness of fit *S* are based on *F*^2^, conventional *R*-factors *R* are based on *F*, with *F* set to zero for negative *F*^2^. The threshold expression of *F*^2^ > 2σ(*F*^2^) is used only for calculating *R*-factors(gt) *etc*. and is not relevant to the choice of reflections for refinement. *R*-factors based on *F*^2^ are statistically about twice as large as those based on *F*, and *R*- factors based on ALL data will be even larger.

### Fractional atomic coordinates and isotropic or equivalent isotropic displacement parameters (Å^2^)

**Table:**

	*x*	*y*	*z*	*U*_iso_*/*U*_eq_	
F4	0.80372 (8)	0.08837 (10)	−0.40649 (7)	0.0903 (3)	
N1	0.26200 (8)	0.23531 (9)	−0.01235 (8)	0.0437 (3)	
N3	0.42488 (8)	0.27540 (10)	0.08546 (8)	0.0497 (3)	
C2	0.39877 (10)	0.23282 (11)	−0.01679 (10)	0.0459 (3)	
C4	0.30225 (10)	0.30605 (11)	0.15929 (9)	0.0455 (3)	
C5	0.19960 (10)	0.28135 (11)	0.10135 (9)	0.0429 (3)	
C11	0.20194 (9)	0.18684 (11)	−0.10385 (9)	0.0439 (3)	
C12	0.16843 (11)	0.07116 (13)	−0.06763 (11)	0.0556 (4)	
C13	0.11969 (13)	0.01955 (16)	−0.15797 (15)	0.0743 (5)	
C14	0.10664 (14)	0.08323 (18)	−0.28296 (15)	0.0794 (6)	
C15	0.14007 (13)	0.19852 (16)	−0.31824 (12)	0.0715 (5)	
C16	0.18572 (11)	0.25319 (13)	−0.22810 (11)	0.0560 (4)	
C21	0.50330 (10)	0.19099 (12)	−0.12073 (10)	0.0477 (3)	
C22	0.51945 (10)	0.08114 (12)	−0.18562 (11)	0.0528 (4)	
C23	0.62124 (11)	0.04652 (13)	−0.28141 (11)	0.0590 (4)	
C24	0.70603 (12)	0.12091 (15)	−0.30998 (11)	0.0625 (4)	
C25	0.69616 (13)	0.22763 (16)	−0.24730 (12)	0.0694 (5)	
C26	0.59330 (12)	0.26275 (14)	−0.15231 (11)	0.0607 (4)	
C41	0.30110 (11)	0.35537 (12)	0.28205 (10)	0.0468 (3)	
C42	0.42822 (12)	0.30638 (14)	0.34878 (11)	0.0602 (4)	
C43	0.43495 (14)	0.35356 (16)	0.46073 (12)	0.0713 (5)	
C44	0.31579 (15)	0.44905 (16)	0.50961 (12)	0.0688 (5)	
C45	0.19033 (14)	0.49726 (14)	0.44586 (12)	0.0654 (5)	
C46	0.18239 (12)	0.45176 (12)	0.33263 (11)	0.0554 (4)	
C51	0.05103 (10)	0.30008 (11)	0.13802 (10)	0.0438 (3)	
C52	0.01739 (12)	0.25395 (13)	0.26117 (11)	0.0549 (4)	
C53	−0.12281 (14)	0.27662 (14)	0.29629 (13)	0.0693 (5)	
C54	−0.23054 (13)	0.34450 (15)	0.20932 (15)	0.0724 (5)	
C55	−0.19831 (11)	0.38917 (14)	0.08727 (13)	0.0638 (4)	
C56	−0.05946 (10)	0.36777 (12)	0.05109 (11)	0.0510 (4)	
H12	0.17852	0.02828	0.01676	0.0667*	
H13	0.09574	−0.05792	−0.13455	0.0892*	
H14	0.07489	0.04771	−0.34393	0.0952*	
H15	0.13200	0.24004	−0.40303	0.0857*	
H16	0.20515	0.33333	−0.25085	0.0672*	
H22	0.46150	0.03061	−0.16453	0.0633*	
H23	0.63160	−0.02634	−0.32557	0.0708*	
H25	0.75675	0.27545	−0.26776	0.0832*	
H26	0.58432	0.33573	−0.10885	0.0728*	
H42	0.50945	0.24100	0.31717	0.0722*	
H43	0.52075	0.32058	0.50362	0.0856*	
H44	0.32052	0.48059	0.58545	0.0825*	
H45	0.10930	0.56144	0.47900	0.0785*	
H46	0.09631	0.48633	0.29000	0.0664*	
H52	0.08926	0.20754	0.32054	0.0659*	
H53	−0.14438	0.24575	0.37930	0.0831*	
H54	−0.32448	0.35981	0.23351	0.0868*	
H55	−0.27068	0.43437	0.02817	0.0766*	
H56	−0.03911	0.39881	−0.03226	0.0612*	

### Atomic displacement parameters (Å^2^)

**Table:**

	*U*^11^	*U*^22^	*U*^33^	*U*^12^	*U*^13^	*U*^23^
F4	0.0761 (5)	0.1160 (7)	0.0732 (5)	−0.0356 (5)	0.0285 (4)	−0.0132 (5)
N1	0.0411 (4)	0.0500 (5)	0.0453 (5)	−0.0228 (4)	0.0001 (3)	−0.0076 (4)
N3	0.0443 (4)	0.0584 (6)	0.0520 (5)	−0.0254 (4)	−0.0013 (4)	−0.0083 (4)
C2	0.0411 (5)	0.0509 (7)	0.0499 (6)	−0.0229 (5)	0.0000 (4)	−0.0051 (5)
C4	0.0445 (5)	0.0491 (6)	0.0468 (6)	−0.0223 (5)	−0.0025 (4)	−0.0051 (5)
C5	0.0426 (5)	0.0447 (6)	0.0442 (5)	−0.0206 (4)	0.0001 (4)	−0.0046 (5)
C11	0.0387 (5)	0.0495 (6)	0.0472 (6)	−0.0196 (5)	0.0009 (4)	−0.0107 (5)
C12	0.0570 (6)	0.0552 (7)	0.0621 (7)	−0.0296 (6)	0.0038 (5)	−0.0103 (6)
C13	0.0762 (8)	0.0745 (9)	0.0928 (11)	−0.0446 (7)	0.0064 (7)	−0.0319 (8)
C14	0.0734 (8)	0.0955 (12)	0.0841 (10)	−0.0365 (8)	−0.0108 (7)	−0.0387 (9)
C15	0.0701 (8)	0.0869 (10)	0.0561 (8)	−0.0245 (8)	−0.0131 (6)	−0.0144 (7)
C16	0.0571 (6)	0.0572 (7)	0.0551 (7)	−0.0235 (6)	−0.0057 (5)	−0.0050 (5)
C21	0.0404 (5)	0.0545 (7)	0.0494 (6)	−0.0210 (5)	−0.0011 (4)	−0.0027 (5)
C22	0.0437 (5)	0.0523 (7)	0.0614 (7)	−0.0188 (5)	0.0039 (5)	−0.0062 (5)
C23	0.0524 (6)	0.0580 (8)	0.0604 (7)	−0.0144 (6)	0.0025 (5)	−0.0091 (6)
C24	0.0487 (6)	0.0770 (9)	0.0532 (7)	−0.0191 (6)	0.0092 (5)	−0.0017 (6)
C25	0.0587 (7)	0.0863 (10)	0.0723 (8)	−0.0427 (7)	0.0096 (6)	−0.0028 (7)
C26	0.0563 (6)	0.0712 (8)	0.0653 (7)	−0.0360 (6)	0.0062 (5)	−0.0127 (6)
C41	0.0512 (6)	0.0504 (6)	0.0466 (6)	−0.0287 (5)	−0.0033 (4)	−0.0032 (5)
C42	0.0540 (6)	0.0720 (9)	0.0595 (7)	−0.0282 (6)	−0.0073 (5)	−0.0093 (6)
C43	0.0750 (8)	0.0885 (10)	0.0608 (8)	−0.0413 (8)	−0.0212 (6)	−0.0043 (7)
C44	0.0920 (9)	0.0822 (10)	0.0509 (7)	−0.0515 (8)	−0.0007 (7)	−0.0146 (7)
C45	0.0749 (8)	0.0694 (9)	0.0625 (7)	−0.0356 (7)	0.0050 (6)	−0.0209 (6)
C46	0.0563 (6)	0.0572 (7)	0.0580 (7)	−0.0255 (6)	−0.0053 (5)	−0.0112 (6)
C51	0.0442 (5)	0.0435 (6)	0.0509 (6)	−0.0231 (5)	0.0041 (4)	−0.0125 (5)
C52	0.0611 (6)	0.0548 (7)	0.0552 (7)	−0.0291 (6)	0.0088 (5)	−0.0117 (5)
C53	0.0808 (9)	0.0690 (9)	0.0711 (8)	−0.0434 (7)	0.0320 (7)	−0.0235 (7)
C54	0.0520 (7)	0.0751 (9)	0.1043 (11)	−0.0360 (7)	0.0224 (7)	−0.0331 (8)
C55	0.0454 (6)	0.0611 (8)	0.0917 (9)	−0.0240 (6)	−0.0002 (6)	−0.0207 (7)
C56	0.0470 (6)	0.0503 (7)	0.0620 (7)	−0.0247 (5)	−0.0009 (5)	−0.0095 (5)

### Geometric parameters (Å, °)

**Table:**

F4—C24	1.3626 (15)	C45—C46	1.3822 (18)
N1—C2	1.3798 (14)	C51—C52	1.3859 (16)
N1—C5	1.3905 (13)	C51—C56	1.3960 (16)
N1—C11	1.4370 (13)	C52—C53	1.386 (2)
N3—C2	1.3194 (14)	C53—C54	1.378 (2)
N3—C4	1.3798 (14)	C54—C55	1.367 (2)
C2—C21	1.4718 (15)	C55—C56	1.3779 (17)
C4—C5	1.3759 (15)	C12—H12	0.9300
C4—C41	1.4761 (15)	C13—H13	0.9300
C5—C51	1.4785 (16)	C14—H14	0.9300
C11—C12	1.3771 (16)	C15—H15	0.9300
C11—C16	1.3793 (15)	C16—H16	0.9300
C12—C13	1.381 (2)	C22—H22	0.9300
C13—C14	1.378 (2)	C23—H23	0.9300
C14—C15	1.372 (2)	C25—H25	0.9300
C15—C16	1.3825 (19)	C26—H26	0.9300
C21—C22	1.3897 (16)	C42—H42	0.9300
C21—C26	1.3887 (18)	C43—H43	0.9300
C22—C23	1.3834 (17)	C44—H44	0.9300
C23—C24	1.3621 (19)	C45—H45	0.9300
C24—C25	1.363 (2)	C46—H46	0.9300
C25—C26	1.3843 (19)	C52—H52	0.9300
C41—C42	1.3950 (18)	C53—H53	0.9300
C41—C46	1.3831 (17)	C54—H54	0.9300
C42—C43	1.3755 (18)	C55—H55	0.9300
C43—C44	1.374 (2)	C56—H56	0.9300
C44—C45	1.365 (2)		
			
F4···H14^i^	2.7400	C42···H54^i^	2.9300
F4···H53^ii^	2.7400	C44···H16^vi^	2.9800
N1···H22	2.9400	C45···H25^vii^	3.0000
N1···H56	2.8800	C46···H52	3.0900
N3···H26	2.6800	C51···H12	3.1000
N3···H42	2.5900	C51···H46	2.9100
N3···H55^iii^	2.9400	C52···H46	2.9200
C2···C22^iv^	3.4772 (16)	C53···H44^viii^	2.9800
C4···C23^iv^	3.5258 (17)	C53···H13^ix^	3.0100
C5···C56^iii^	3.5569 (16)	C54···H13^ix^	3.0100
C5···C23^iv^	3.5260 (16)	C54···H44^viii^	2.9400
C11···C56	3.1419 (16)	C56···H56^iii^	2.9700
C11···C22	3.0928 (15)	H12···C5	3.0300
C12···C51	3.3300 (16)	H12···C51	3.1000
C12···C56	3.4599 (17)	H13···C53^ix^	3.0100
C16···C21	3.2942 (17)	H13···C54^ix^	3.0100
C16···C22	3.2012 (17)	H14···F4^x^	2.7400
C21···C16	3.2942 (17)	H16···C2	3.0700
C22···C11	3.0928 (15)	H16···C44^v^	2.9800
C22···C2^iv^	3.4772 (16)	H22···N1	2.9400
C22···C16	3.2012 (17)	H22···C11	2.6200
C23···C4^iv^	3.5258 (17)	H22···C12	2.9700
C23···C5^iv^	3.5260 (16)	H22···C16	2.9200
C41···C52	3.4747 (18)	H22···C2^iv^	3.0100
C46···C51	3.4230 (16)	H23···C4^iv^	3.0400
C46···C52	3.3345 (18)	H25···C45^vii^	3.0000
C51···C46	3.4230 (16)	H26···N3	2.6800
C51···C12	3.3300 (16)	H42···N3	2.5900
C52···C46	3.3345 (18)	H42···H54^i^	2.5000
C52···C41	3.4747 (18)	H43···C24^vi^	2.8400
C56···C11	3.1419 (16)	H44···C53^viii^	2.9800
C56···C56^iii^	3.4379 (16)	H44···C54^viii^	2.9400
C56···C5^iii^	3.5569 (16)	H46···C5	3.0200
C56···C12	3.4599 (17)	H46···C51	2.9100
C2···H22^iv^	3.0100	H46···C52	2.9200
C2···H16	3.0700	H52···C4	3.0500
C4···H55^iii^	3.0300	H52···C41	3.0800
C4···H23^iv^	3.0400	H52···C46	3.0900
C4···H52	3.0500	H53···F4^xi^	2.7400
C5···H46	3.0200	H54···C42^x^	2.9300
C5···H12	3.0300	H54···H42^x^	2.5000
C11···H56	2.7600	H55···N3^iii^	2.9400
C11···H22	2.6200	H55···C4^iii^	3.0300
C12···H22	2.9700	H56···N1	2.8800
C16···H22	2.9200	H56···C11	2.7600
C16···H56	3.0900	H56···C16	3.0900
C24···H43^v^	2.8400	H56···C56^iii^	2.9700
C41···H52	3.0800		
			
C2—N1—C5	107.30 (9)	C52—C53—C54	120.60 (13)
C2—N1—C11	125.41 (9)	C53—C54—C55	119.52 (13)
C5—N1—C11	127.07 (9)	C54—C55—C56	120.53 (12)
C2—N3—C4	106.26 (9)	C51—C56—C55	120.81 (11)
N1—C2—N3	110.82 (9)	C11—C12—H12	120.00
N1—C2—C21	125.85 (9)	C13—C12—H12	120.00
N3—C2—C21	123.32 (10)	C12—C13—H13	120.00
N3—C4—C5	110.51 (9)	C14—C13—H13	120.00
N3—C4—C41	118.29 (10)	C13—C14—H14	120.00
C5—C4—C41	131.20 (10)	C15—C14—H14	120.00
N1—C5—C4	105.11 (9)	C14—C15—H15	120.00
N1—C5—C51	122.73 (9)	C16—C15—H15	120.00
C4—C5—C51	132.12 (9)	C11—C16—H16	121.00
N1—C11—C12	119.53 (9)	C15—C16—H16	121.00
N1—C11—C16	119.13 (10)	C21—C22—H22	120.00
C12—C11—C16	121.26 (10)	C23—C22—H22	120.00
C11—C12—C13	119.27 (11)	C22—C23—H23	121.00
C12—C13—C14	119.78 (14)	C24—C23—H23	121.00
C13—C14—C15	120.59 (14)	C24—C25—H25	121.00
C14—C15—C16	120.18 (12)	C26—C25—H25	121.00
C11—C16—C15	118.87 (12)	C21—C26—H26	119.00
C2—C21—C22	123.50 (10)	C25—C26—H26	119.00
C2—C21—C26	117.88 (10)	C41—C42—H42	120.00
C22—C21—C26	118.59 (10)	C43—C42—H42	120.00
C21—C22—C23	120.38 (11)	C42—C43—H43	120.00
C22—C23—C24	118.91 (12)	C44—C43—H43	120.00
F4—C24—C23	118.56 (12)	C43—C44—H44	120.00
F4—C24—C25	118.58 (12)	C45—C44—H44	120.00
C23—C24—C25	122.85 (12)	C44—C45—H45	120.00
C24—C25—C26	118.04 (13)	C46—C45—H45	120.00
C21—C26—C25	121.21 (12)	C41—C46—H46	120.00
C4—C41—C42	118.31 (11)	C45—C46—H46	120.00
C4—C41—C46	123.79 (11)	C51—C52—H52	120.00
C42—C41—C46	117.86 (11)	C53—C52—H52	120.00
C41—C42—C43	120.84 (13)	C52—C53—H53	120.00
C42—C43—C44	120.44 (14)	C54—C53—H53	120.00
C43—C44—C45	119.41 (13)	C53—C54—H54	120.00
C44—C45—C46	120.75 (13)	C55—C54—H54	120.00
C41—C46—C45	120.70 (12)	C54—C55—H55	120.00
C5—C51—C52	120.62 (10)	C56—C55—H55	120.00
C5—C51—C56	121.15 (9)	C51—C56—H56	120.00
C52—C51—C56	118.21 (11)	C55—C56—H56	120.00
C51—C52—C53	120.33 (12)		
			
C5—N1—C2—N3	−0.64 (12)	C16—C11—C12—C13	0.98 (18)
C5—N1—C2—C21	−179.79 (10)	N1—C11—C16—C15	174.19 (11)
C11—N1—C2—N3	−175.57 (9)	C12—C11—C16—C15	−2.50 (18)
C11—N1—C2—C21	5.28 (17)	C11—C12—C13—C14	0.7 (2)
C2—N1—C5—C4	0.72 (11)	C12—C13—C14—C15	−0.8 (2)
C2—N1—C5—C51	178.72 (10)	C13—C14—C15—C16	−0.8 (2)
C11—N1—C5—C4	175.54 (10)	C14—C15—C16—C11	2.4 (2)
C11—N1—C5—C51	−6.46 (16)	C2—C21—C22—C23	−179.20 (11)
C2—N1—C11—C12	112.68 (12)	C26—C21—C22—C23	−1.36 (17)
C2—N1—C11—C16	−64.07 (15)	C2—C21—C26—C25	178.74 (12)
C5—N1—C11—C12	−61.25 (15)	C22—C21—C26—C25	0.78 (18)
C5—N1—C11—C16	122.00 (12)	C21—C22—C23—C24	0.66 (18)
C4—N3—C2—N1	0.28 (12)	C22—C23—C24—F4	−178.45 (11)
C4—N3—C2—C21	179.46 (10)	C22—C23—C24—C25	0.7 (2)
C2—N3—C4—C5	0.20 (12)	F4—C24—C25—C26	177.87 (12)
C2—N3—C4—C41	179.33 (10)	C23—C24—C25—C26	−1.3 (2)
N1—C2—C21—C22	−38.85 (17)	C24—C25—C26—C21	0.5 (2)
N1—C2—C21—C26	143.30 (11)	C4—C41—C42—C43	177.03 (12)
N3—C2—C21—C22	142.10 (12)	C46—C41—C42—C43	−0.48 (19)
N3—C2—C21—C26	−35.75 (16)	C4—C41—C46—C45	−177.52 (12)
N3—C4—C5—N1	−0.57 (12)	C42—C41—C46—C45	−0.15 (18)
N3—C4—C5—C51	−178.31 (11)	C41—C42—C43—C44	0.7 (2)
C41—C4—C5—N1	−179.56 (11)	C42—C43—C44—C45	−0.2 (2)
C41—C4—C5—C51	2.7 (2)	C43—C44—C45—C46	−0.4 (2)
N3—C4—C41—C42	−31.76 (15)	C44—C45—C46—C41	0.6 (2)
N3—C4—C41—C46	145.60 (12)	C5—C51—C52—C53	177.49 (11)
C5—C4—C41—C42	147.16 (13)	C56—C51—C52—C53	−0.79 (18)
C5—C4—C41—C46	−35.48 (19)	C5—C51—C56—C55	−177.69 (11)
N1—C5—C51—C52	135.85 (11)	C52—C51—C56—C55	0.59 (17)
N1—C5—C51—C56	−45.92 (16)	C51—C52—C53—C54	0.4 (2)
C4—C5—C51—C52	−46.76 (18)	C52—C53—C54—C55	0.3 (2)
C4—C5—C51—C56	131.48 (13)	C53—C54—C55—C56	−0.5 (2)
N1—C11—C12—C13	−175.70 (11)	C54—C55—C56—C51	0.1 (2)

Symmetry codes: (i) *x*+1, *y*, *z*; (ii) *x*+1, *y*, *z*−1; (iii) −*x*, −*y*+1, −*z*; (iv) −*x*+1, −*y*, −*z*; (v) *x*, *y*, *z*−1; (vi) *x*, *y*, *z*+1; (vii) −*x*+1, −*y*+1, −*z*; (viii) −*x*, −*y*+1, −*z*+1; (ix) −*x*, −*y*, −*z*; (x) *x*−1, *y*, *z*; (xi) *x*−1, *y*, *z*+1.
